# Risk perception and neuro-emotional development in adolescent athletes: psychometric validation of the ESTUDES scales for preventive guidance contexts

**DOI:** 10.3389/fpsyg.2026.1818238

**Published:** 2026-04-09

**Authors:** Juan Carlos Armenteros Mayoral, Franciso Manuel Raso Sánchez, Álvaro Manuel Úbeda Sánchez, Daniel Álvarez Ferrándiz

**Affiliations:** 1 Pedagogy Department, Universidad de Granada, Granada, Spain; 2 Instituto Nacional de Evaluación Educativa, Madrid, Spain; 3 Didactics and Scholar Organization, Universidad de Granada, Granada, Spain; 4 Pedagogy Department, Universidad de Jaén, Jaén, Spain

**Keywords:** drugs, prevention, sport, validation, young people

## Abstract

**Introduction:**

Organized sport promotes physical and psychological health in adolescents, improving emotional regulation, self-efficacy and resilience, which are essential for preventing addiction. This study validates an adapted ESTUDES scale for measuring consumption and risk perception in young athletes.

**Method:**

Descriptive cross-sectional study with 914 adolescent footballers from Jaén and Granada (aged 12–18). The ESTUDES 2023 questionnaire (20 items on tobacco, alcohol, cannabis, gambling, and risk perception) was adapted, applying exploratory and confirmatory factor analysis.

**Results:**

Five factors were identified, explaining 73.47% of the variance: tobacco use, alcohol use, gambling, cannabis use, and risk perceptions. Reliability was adequate (*α* = 0.863) with satisfactory fit indices (CFI = 0.920; RMSEA = 0.060). The highest correlations appeared between tobacco and alcohol (0.564), while risk perceptions showed negative relationships with all types of consumption.

**Conclusion:**

The scale demonstrated adequate validity and consistency, constituting a useful tool for assessing addictive behaviors and designing preventive programs for adolescent athletes.

## 1 Introduction

Sports practices constitute a set of positive behaviors towards health that result in a better quality of life and psychological wellbeing (González Moreno and Molero Jurado, 2024), especially when we talk about a structured context such as sport, which shows greater benefits than recreational physical activity (Wilson et al., 2022). Such benefits can include interpersonal aspects such as improved socialization or the absence of stereotypes in populations with disabilities (Aitchison et al., 2022), or intrapersonal aspects such as emotion regulation (Muñoz-Villena et al., 2020; Portela-Pino et al., 2024; Šagát et al., 2021). All these aspects protect adolescents from stressful everyday situations such as the search for a secure professional future or the improvement of academic performance, i.e., a context marked by the autonomy characteristic of this stage (Constantinou and Aguiyi, 2022; Carton et al., 2023). To avoid these challenging situations, skills such as self-efficacy and personal resilience are required, which enable the management of adverse events such as interpersonal and academic stress related to the increase in symptoms of mental disorders (Rayburn et al., 2021; Yang et al., 2023).

The development of the aforementioned skills is acquired thanks to the effects of neuroplasticity induced by physical activity in individual brain regions and largescale neural circuits, manifesting itself in the optimization of stress reactivity and mitigating the effects of stressful events (Belcher et al., 2021; Arida and Teixeira-Machado, 2021; Neumann et al., 2022). It is during adolescence that these emotional regulation skills are essential. These abilities allow individuals to balance short-term individual interests with long-term collective benefits or, as Bauman (2007) points out, “social hierarchy is measured by the increasing ability to get what one want” (p. 22).

In contemporary youth contexts, promoting universal prevention strategies that prevent, or limit substance abuse and behavioral addictions has become a priority for public policy. Personal, social and contextual skills are priority objectives for reducing vulnerability to drug use and other behaviors that can lead to addiction, in addition to promoting healthy lifestyles and healthy leisure alternatives that are incompatible with substance abuse and other addictive behaviors (Ministerio de Sanidad, Consumo y Bienestar Social, 2018). This study is part of this line of thinking, focusing on the benefits of healthy habits and leisure alternatives such as sport and their influence on addiction through the application of the Uso de Drogas en Enseñanzas Secundarias en España (ESTUDES). Thus, the study aims to provide a descriptive overview of addictive consumption among adolescent athletes, but also to contribute to the debate on the various prevention programs for addictive behaviors.

The scale used in the research is adapted to the context and sample of the ESTUDES survey. The scale consists of 34 items corresponding to the variables of interest for addressing consumption and risk perception regarding addictions (Ministerio de Sanidad, 2023). Specifically, consumption is measured by age of onset and lifetime, annual and monthly frequency of consumption, while risk perception is quantified by opinion on the social and health conflicts involved in engaging in different addictive behaviors.

Other instruments, such as the AUDIT questionnaire, are designed for clinical screening and are not intended to monitor specific contexts or patterns (Horváth et al., 2023). That is why the instruments take into account theories such as Bandura’s (1977) social learning theory, which offer explanatory frameworks on the aetiology of adolescent consumption, where variables such as peer pressure in sports or team culture play a differential role.

The analysis of addictive behaviors is a key indicator for preventing harm to both users and society. Various theorists point out that adolescence is a stage of life when individuals are more likely to develop addictive behaviors, resulting in personal harm such as mortality, dependence, loss of possessions and relationships, among others (Steinberg, 2022; Nutt et al., 2010). These persistent addictive behaviors over time cause changes in different brain receptors that affect brain processing, such as the metabolization of ethanol in NMDA receptors, the main excitatory neurotransmitter in the human brain that is present in processes such as memory or in pathophysiology such as epilepsy or Alzheimer’s disease (Jewett and Thapa, 2025). For this reason, the main objective of the present study was to validate a scale that accurately measures consumption and thinking about addiction in young people in protective contexts, in order to obtain a robust instrument that facilitates this assessment. The secondary objective was to facilitate decision making for the development of specific prevention programs in sports contexts. This tool will provide a concise, rigorous and comparable assessment of addiction consumption in relation to the average adolescent.

## 2 Materials and methods

This study is descriptive, exploratory, and cross-sectional in nature. A sample of 914 adolescent football and futsal players from Andalusia was obtained (Jaén capital = 28%, Granada capital = 45.6%, and Granada province = 26.4%). The age distribution consisted of 304 (33.3%) players in the under13s category (1,213 years old), 364 (39.3%) players in the under 15s category (1,415 years old) and 246 (26.9%) players in the under 18s category (1,618 years old).

Most of these young people come from families where at least one of their parents is employed. In terms of educational level, mothers have a higher level of university education than fathers (40.8 and 35.6%, respectively). In terms of economic status, most participants (82.9%) perceive themselves to have a similar economic status to that of their context. Finally, the amount of money available for going out is represented in a U shape, with the most common amounts being €0–5 (28.7%) and more than €15 (21.9%).

This research complied with the standards of the favorable opinion of the Human Research Committee of the University of Granada (No. 4968/CEIH/2025) and the ethical principles established in the Declaration of Helsinki (1975).

### 2.1 Instrument

The questionnaire used for the Ministerio de Sanidad (2023) ESTUDES 2023 report was employed, which is standardized and similar to other surveys conducted in Europe and the United States. The questionnaire used by the Ministry of Health is composed of different modules that have been added over the years.

This study draws on two specific modules. First, the basic module assesses sociodemographic data on students, their families, their leisure habits and consumption patterns of alcohol, tobacco and cannabis consumption, alongside the perception of risk of addictive behaviors. This module was selected because these substances represent the most prevalent and empirically documented substances in adolescent athletic populations (Ministerio de Sanidad, 2023). From the module added in 2014 with topics related to gambling, questions on the same topic as alcohol, tobacco and cannabis consumption were selected.

Remaining modules were excluded as their prevalence and theoretical relevance in structured sports contexts remains insufficiently established to warrant inclusion in an initial validation study.

### 2.2 Procedure

To obtain data for the study, clubs were randomly selected, followed by the teams that make up those clubs, and the questionnaire was given to all players present. Contact was made by means of an information document detailing the objectives of the study, the commitment to return the overall results obtained, and to ensure confidentiality and anonymity throughout the process.

After receiving authorization from the club management or youth team coordinators, informed consent was requested from the legal guardians of the potential participants. The instruments were then administered during the last 4 months of 2024. The questionnaires were administered in the presence of a researcher and the coach of the corresponding team, in order to ensure that the instruments were completed correctly and to resolve any potential queries. The entire procedure was carried out under normal conditions and without any notable incidents.

It should be noted that the anonymity of the participants was always guaranteed, with the responses to the questionnaires being anonymous and the participants’ rights to confidentiality being respected at all times.

### 2.3 Data analysis

To perform the basic descriptive analyses corresponding to exploratory factor analysis (EFA), IBM SPSS® software version 22.0 and JASP 0.95.4 software were used, while factor loadings and the rotated matrix were obtained using JASP 0.95.4 software. With regard to EFA, the maximum likelihood method with varimax rotation was used (excluding factor loading of less than 0.4), and Cronbach’s alpha was calculated to determine the internal consistency of the scale (95% reliability index). Confirmatory factor analysis (CFA) used goodness of fit indices and Chi-square tests. The variables were not normally distributed and were ordinal in nature; the estimates did not depend on any assumptions about the distribution of the items. To ensure that the observed kurtosis did not affect the standard errors, the diagonalized weighted least squares (DWLS) adjustment function was used (Romero and Babativa, 2016).

To assess the adequacy of the data, the Kaiser Meyer Olkin (KMO) measure was calculated, in which an index is satisfactory if it is greater than 0.80 (Kaiser, 1970). To establish the model fit, the root mean square error of approximation (RMSEA), standardized root mean square residual (SRMR), comparative fit indices (CFI), normalized fit index (NFI) and incremental fit index (IFI) were considered. The indices representing a good fit between the model and the data are RMSEA < 0.06, SRMR < 0.08, CFI > 0.95, and TLI > 0.95 (Xia and Yang, 2018, 2019).

## 3 Results

Table 1 shows the description of the items, codes, values, and basic descriptive statistics corresponding to the 20 items that make up the addictive consumption scale. As can be seen, the questionnaire was developed based on the structure and dimensions established by the basic module included in all years of the ESTUDES survey and a module on gambling that was added in 2014, developing an abbreviated version of that scale.

**Table 1:** Characteristics and properties of items.

Items	Code	Value	Frequency	Percent
How old were you when you first used tobacco?	ATI	Never	81048	88.6
		0–7 years	8	0.9
		8–14 years	48	5.3
		15 years or older	48	5.3
How old were you when you first used alcohol?	AAI	Never	585	64.0
		0–7 years	42	4.6
		8–14 years	154	16.8
		15 years or older	133	14.6
How old were you when you first used cannabis?	ACI	Never	858	93.9
		0–7 years	9	1.0
		8–14 years	13	1.4
		15 years or older	34	3.7
How old were you when you first gambled?	AGI	Never	662	72.4
		0–7 years	51	5.6
		8–14 years	85	9.3
		15 years or older	116	12.7
How many times have you used cigarettes in your life?	LTF	0–3 times	851	93.1
		4–9 times	14	1.5
		10–39 times	22	2.4
		More than 39 times	27	3.0
How many times have you used cigarettes in the last 12 months?	ATF	0–3 times	860	94.1
		4–9 times	22	2.4
		10–39 times	19	2.1
		More than 39 times	13	1.4
How many times have you used cigarettes in the last 30 days?	MTF	0–3 times	878	96.1
		4–9 times	20	2.2
		10–39 times	7	0.8
		More than 39 times	9	1.0
How many times have you used alcohol in your life?	LAF	0–3 times	643	70.4
		4–9 times	55	6.0
		10–39 times	87	9.5
		More than 39 times	129	14.1
How many times have you used alcohol in the last 12 months?	AAF	0–3 times	677	74.1
		4–9 times	84	9.2
		10–39 times	82	9.0
		More than 39 times	71	7.8
How many times have you used alcohol in the last 30 days?	MAF	0–3 times	785	85.9
		4–9 times	71	7.8
		10–39 times	33	3.6
		More than 39 times	25	2.7
How many times have you used cannabis in your life?	LCF	0–3 times	892	97.6
		4–9 times	10	1.1
		10–39 times	5	0.5
		More than 39 times	7	0.8
How many times have you used cannabis in the last 12 months?	ACF	0–3 times	901	98.6
		4–9 times	6	0.7
		10–39 times	6	0.7
		More than 39 times	1	0.1
How many times have you used cannabis in the last 30 days?	MCF	0–3 times	905	99.0
		4–9 times	8	0.9
		10–39 times	1	0.1
		More than 39 times	0	0
How many times have you gambled in your life?	LGF	0–3 times	720	78.8
		4–9 times	68	7.4
		10–39 times	67	7.3
		More than 39 times	59	6.5
How many times have you gambled in the last 12 months?	AGF	0–3 times	752	82.3
		4–9 times	86	9.4
		10–39 times	47	5.1
		More than 39 times	29	3.2
How many times have you gambled in the last 30 days?	MGF	0–3 times	809	88.5
		4–9 times	64	7.0
		10–39 times	22	2.4
		More than 39 times	19	2.1
What is your opinion on the problems that smoking a pack a day can cause?	TPR	No or few problems	89	9.7
		Some or many problems	825	90.3
What is your opinion on the problems that smoking 1 to 5 cigarettes a day can cause?	DCR	No or few problems	168	18.4
		Some or many problems	746	81.6
What is your opinion on the problems that drinking 5 or 6 alcoholic beverages on the weekend can cause?	WAR	No or few problems	186	20.4
		Some or many problems	728	79.6
What is your opinion on the problems that drinking 1 or 2 alcoholic beverages every day can cause?	DAR	No or few problems	288	31.5
		Some or many problems	626	68.5

Subsequently, the sedimentation graph is drawn up with the aim of verifying the ideal number of factors for the factorial solution according to Kaiser’s criterion (components with eigenvalues greater than 1) and the “elbow” criterion (inflection point of the curve). It was observed that the first component shows a significantly higher eigenvalue than the rest of the components (greater than 6), followed by a sharp drop. From the second component onwards, the values tend to homogenize, showing an “elbow” between components 4 and 5.

Table 2 shows the results regarding the psychometric properties of the ESTUDES questionnaire when only the 16 items related to the consumption of the main addictions are considered. A rotated factor matrix was used, together with factor loadings using the JASP 0.95.4 program through principal components and varimax rotation.

**Table 2:** Rotated factorial matrix and factorial loadings.

Code	Factor 1	Factor 2	Factor 3	Factor 4	Factor 5	Uniqueness
ATF	0.968					0.005
LTF	0.889					0.109
MTF	0.838					0.253
ATI	0.559					0.536
AGF		0.954				0.023
LGF		0.861				0.186
MGF		0.808				0.294
AGI		0.638				0.504
LAF			0.937			0.041
AAF			0.873			0.144
AAI			0.733			0.423
MAF			0.501			0.548
ACF				0.985		0.005
MCF				0.865		0.225
LCF				0.813		0.250
DCR					0.714	0.487
DAR					0.656	0.553
TPR					0.516	0.698
WAR					0.432	0.810
ACI				0.335		
Eigenvalue	6.427	2.301	1.895	1.427	1.241	—
% explained variance	33.43%	12.43%	10.43%	9.79%	7.39%	73.47%
Reliability *α*	0.881	0.890	0.879	0.849	0.648	0,863

The correlation matrix was adequate, as Bartlett’s statistic showed a good fit [14685.749; gl = 190; *p* < 0.001]. This indicates the absence of an identity matrix. The Kaiser-Meyer-Olkin (KMO) index value was good [KMO = 0.811] and is consistent with Bartlett’s sphericity index, since values close to one indicate that the quadratic sums of partial correlations is weak, while correlations between items are more notable.

It should be noted that the determinant of the matrix reaches a value |*R*| of 0.00000009046. This implies that there is a sufficient relationship between the variables to be able to extract factors, without falling into complete collinearity. This aspect is also essential, as it implies that the matrix is invertible, which is necessary for EFA, especially when performing extraction procedures.

The EFA presented five factors, which explained 73.47% of the total variance, which is an adequate percentage. The distribution in Table 2 is presented in descending order, and those below 0.40 are not considered.

Prior to interpreting the factors obtained, the results will be presented in relation to the eigenvalues, the variance explained by each factor, and their contribution to the overall factor structure.

Through EFA, five eigenvalues greater than one were extracted, in accordance with Kaiser’s criterion. The first factor had an eigenvalue of 6.427, demonstrating its dominance in comparison with the other factors. Factors 2, 3, 4, and 5 obtained eigenvalues of 2.301, 1.895, 1.427, and 1.241, respectively. After applying orthogonal rotation, the variance was redistributed in a more balanced way, obtaining explained variances of 33.43%, 12.43%, 10.43%, 9.79%, and 7.39%, adding up to a total of 73.47% of explained variance.

The first factor continues to stand out for its greater explanatory power, accompanied by a large reduction in the eigenvalues in the remaining four factors, which may suggest a defined factor structure. However, the improvement in the distribution after rotation supports the factorial interpretation, being above 60%, which is usually considered good in the social sciences.

Factor 1 is interpreted as “tobacco use”, grouping together the items on age of onset of tobacco use and frequency of use in lifetime, in the last 12 months and in the last 30 days. The same applies to factors 2 and 3, which are interpreted as “gambling” and “alcohol use”, respectively. Factors 2 and 3 have the same variables as factor 1, but with the aforementioned addiction theme.

Factor 4, interpreted as “cannabis use”, shows a pattern similar to factors 1, 2, and 3, but excludes the variable of age of onset of cannabis use because it has a factor loading of less than 0.4.

Finally, factor 5 is defined as ‘perceptions of risk in addictive behaviors’ and consists of four items. This factor focuses on information about the individual biological and social dangers of tobacco and alcohol consumption in different doses.

Furthermore, it should be noted that the reliability indices obtained for almost all dimensions are satisfactory. Factor 2 has the highest internal consistency (*α* = 0.890), followed by factor 1 (*α* = 0.881) and then factor 3 (*α* = 0.879), indicating a solid homogeneity of the items. Factor 4 also showed adequate values (*α* = 0.849). However, factor 5 presented slightly lower values (*α* = 0.648), although it also reaches acceptable levels of reliability. Overall, the total scale shows acceptable reliability (*α* = 0.863).

A confirmatory factor analysis was then performed to corroborate the consistency values of the scale obtained through EFA. To this end, a structural equation model was constructed that included the different factors and grouped items from the previous analysis. The model comprised a total of five factors and a total of 19 observed variables corresponding to the items included in the final scale.

The model fit shows a chi-square value with a significant *p*-value (*χ*^2^ = 610.752; d*f* = 142; *p* < 0.001). However, this measure is sensitive to sample size, so it was decided to use other fit indices. Thus, the incremental fit index (IFI), normed fit index (NFI), comparative fit index (CFI) and Tucker–Lewis index (TLI) showed near acceptable values in IFI and CFI (0.920 in both cases), as did the NFI (0.900) and TLI (0.904). On the other hand, the root mean square error of approximation (RMSEA) value was adequate, standing at 0.060, while the standardized root mean square residual (SRMR) was acceptable, with a value of 0.059.

Table 3 presents the results of the standardized factor loadings obtained through confirmatory factor analysis. We found a wide range of results in the estimates, establishing 15 variables with high estimators (>0.7) and four variables with moderate-high estimators (0.5–0.7). However, all variables show statistically significant values (*p* < 0.001).

**Table 3:** Factor loadings obtained through confirmatory factor analysis.

Factor	Indicator	Estimate	Std. error	*z*-value	*p*	95% confidence interval	
						Lower	Upper
Factor 1	LTF	0.977	0.017	56.45	<0.001	0.943	1.011
	ATF	0.905	0.013	69.53	<0.001	0.880	0.931
	MTF	0.795	0.016	50.78	<0.001	0.764	0.825
	ATI	0.768	0.020	38.65	<0.001	0.730	0.807
Factor 2	AGI	0.743	0.031	24.24	<0.001	0.683	0.803
	LGF	0.913	0.021	43.02	<0.001	0.871	0.954
	AGF	0.933	0.014	68.64	<0.001	0.906	0.960
	MGF	0.826	0.016	50.65	<0.001	0.794	0.858
Factor 3	AAI	0.605	0.035	17.41	<0.001	0.537	0.673
	AAF	0.798	0.024	32.97	<0.001	0.750	0.845
	LAF	0.808	0.029	28.13	<0.001	0.751	0.864
	MAF	0.864	0.022	39.34	<0.001	0.821	0.907
Factor 4	LCF	0.934	0.008	121.55	<0.001	0.919	0.949
	ACF	0.956	0.006	162.23	<0.001	0.945	0.968
	MCF	0.817	0.008	108.90	<0.001	0.803	0.832
Factor 5	TPR	0.589	0.030	19.51	<0.001	0.529	0.648
	DCR	0.711	0.036	19.86	<0.001	0.641	0.781
	WAR	0.574	0.036	16.09	<0.001	0.504	0.644
	DAR	0.544	0.050	10.95	<0.001	0.447	0.642

Within factor 1 (tobacco consumption), the highest loading corresponds to the LTF item (0.977), while the lowest is observed in the ATI item (0.768). In the case of factor 2 (gambling), the highest loading corresponds to the AGF item (0.933), while the lowest belongs to the AGI item (0.743). For factor 3 (alcohol consumption), the MAF item has the highest loading (0.864), while the AAI item has the lowest (0.605). With regard to factor 4 (cannabis use), the highest factor loading corresponds to the ACF item (0.956), while the lowest is associated with the MCF item (0.817). Finally, with regard to factor 5 (perceptions of risk in addictive behaviors), the highest loading corresponds to the DCR item (0.711), while the lowest is observed in the TPR item (0.544).

The results obtained confirm that the dimensions of factors 1, 2, 3, and 5 are supported by the items that comprise them. However, factor 5 (perceptions of risk in addictive behaviors) has notably less factor loadings.

The standardized covariance values between factors reveal statistically significant relationships (*p* < 0.001), suggesting that the factor structure is integrated and consistent. The highest positive correlation was obtained between factor 1 (tobacco use) and factor 3 (alcohol use), with a coefficient of 0.564, reflecting a strong theoretical link between the comorbidity of both substances. The lowest positive correlation was observed between factor 3 (alcohol consumption) and factor 4 (cannabis consumption), with a value of 0.323, which could be interpreted as a differentiation between different patterns of consumption. One of the most relevant findings is that factor 5 (perceptions of risk in addictive behaviors) shows negative correlations with all other factors related to consumption, the most pronounced being with factor 3 (alcohol consumption) and factor 2 (gambling), with coefficients of −0.227 and −0.152, respectively, indicating that a greater perception of risk is associated with lower levels of addictive behaviors.

**Figure 1:**
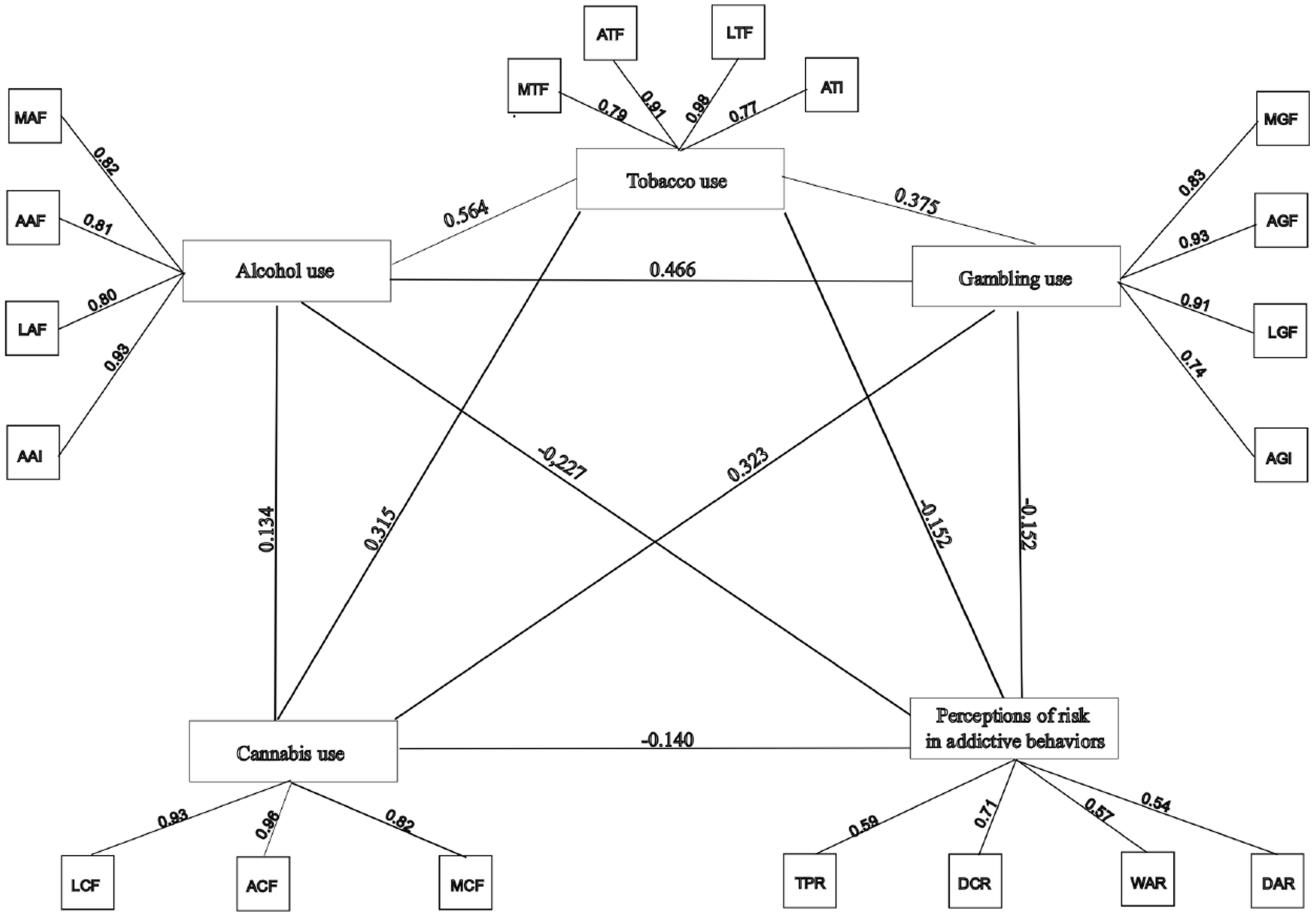
Structural model for confirmatory factor analysis.

The visual representation of the structural model provides graphic confirmation of the adequacy and robustness of the identified five-factor model (Figure 1). The paths linking the items to their factors’ show differentiated loadings consistent with the results obtained previously. Furthermore, the correlations between factors, represented by bidirectional arrows at the top of the diagram, are consistent with the existing literature on addictive behaviors and risk perception in adolescents. Of particular note is the negative relationship between factor 5 (perceptions of risk in addictive behaviors) and consumption factors, as well as the positive correlations between different types of consumption, particularly between tobacco and alcohol. Overall, the model is statistically robust, theoretically consistent, and visually clear. This suggests that the scale is structurally valid and useful as a multidimensional assessment tool.

## 4 Discussion

The aim of this research was to validate a scale for measuring consumption and perceptions of behaviors related to tobacco, alcohol and cannabis among adolescent athletes using exploratory and confirmatory factor analysis. The relevance of this study is based on the need for valid and reliable instruments that allow for a comprehensive comparison of adolescent addictive consumption in different contexts. In this sense, the scale seeks to contribute to filling a gap in the health, educational and social fields, as it adapts a tool originally designed for use in school contexts among 14- to 18-year-olds and reconstructs it for application to younger ages (from 12 years old) and sporting contexts. This research is similar to recent previous studies conducted on both trainers (López Muñiz et al., 2024) and athletes (Alexe et al., 2022; Trigueros et al., 2021).

EFA identified a structure composed of five clearly differentiated factors. No previous data from the Ministry of Health has been found on the different modules that make up an EFA. However, a study by Méndez Mateo et al. (2017) has been found in the scientific literature, which uses a previous version of the ESTUDES report and obtains two dimensions based on legal and illegal drugs. This finding demonstrates the richness of the items used on consumption and perception of various addictions. The statistical suitability of the proposal is supported by outstanding KMO, Bartlett’s and internal consistency indices (Cronbach’s alpha). In addition, the total variance explained exceeded 50%. All of the above statistics reveal that the model is robust (Bonett and Wright, 2015; Marsh et al., 2020).

At a theoretical level, the identified dimensions show a clear thematic connection. The first factor “tobacco use,” integrates elements of age of onset of use and lifetime, annual, and monthly frequency of tobacco use. The second factor, “gambling,” the third factor, “alcohol use,” and the fourth factor, “cannabis use,” group the same items as factor one, but refer to the thematic addiction. Finally, the fifth factor “perceptions of risk in addictive behaviors”, synthesizes the items that relate to the social and health dangers that can result from certain behaviors related to addictive consumption.

Regarding the fifth factor, “perceptions of risk in addictive behaviors,” it is recognized that the four items that make up this dimension have somewhat lower internal consistency (*α* = 0.648) compared to the other factors. Although this value remains within an acceptable range for exploratory instruments (Nunnaly, 1978), it is recognized that four items may not fully capture the complexity of risk perception as a multidimensional construct. Future versions of the instrument should consider expanding this dimension with additional items.

The confirmatory factor analysis recognized the five-factor structure, obtaining high factor loadings in all items. Additionally, all items obtained significance statistics in this analysis. Together, the structural model fit indices (CFI, IFI, NFI, TLI, and RMSEA) are within acceptable ranges, implying structural consistency (Cunningham et al., 2001). However, the chi-square statistic value was significant, a value that is sensitive to the sample size, which implies a cautious interpretation and justifies the need to calculate the aforementioned indices (Morata-Ramirez et al., 2015).

The relationships obtained between the elements included in the model are equally noteworthy, as they suggest a cohesive structure in which all dimensions are connected to each other without losing their uniqueness. The most notable relationships are observed between tobacco, alcohol and gambling consumption, suggesting that young people with addictive consumption develop poly-consumption of legal addictions. This association highlights the importance of working on the prevention of all addictions to avoid the gateway phenomenon to the world of drugs (Nkansah-Amankra and Minelli, 2016; Maldonado-Molina and Lanza, 2010).

It should be noted that sociodemographic data and lifetime frequency of addictive behaviors may lead to responses susceptible to memory recall problems or possible social desirability biases. Although this latter limitation is inherent to administered surveys that include identifying data records.

## 5 Conclusion

The scale developed to measure addictive consumption in young athletes’ demonstrated adequate structural validity and internal consistency through exploratory and confirmatory factor analysis. Based on a rigorous restructuring of the ACI item, a five-factor model was defined that allows for a coherent evaluation of the construction. Specifically, the four factors are: tobacco use, alcohol use, gambling, cannabis use, and perceptions of risk in addictive behaviors.

The results obtained in the goodness-of-fit indices for the confirmatory model, together with the standardized factor loadings, show that the instrument is robust and useful for identifying patterns of consumption in adolescent athletes. Similarly, the identified factor structure reflects consistent consumption patterns that are in line with current mainstream theories of the development of addictive behaviors in young people. It is proposed that this profile be considered in educational and employability policies reflected in regional regulations and initiatives.

The brevity of the survey, together with its conceptual clarity and comparability with national reports, makes it a useful tool for application in youth contexts and prevention programs. The implementation of the scale can contribute both to the identification of the main addictive behaviors in sport and to the evaluation of selective interventions aimed at athletes. Similarly, it is considered imperative to continue with the validation of this scale in larger and more varied samples, while investigating its connection with external variables, such as mental illness, family context and peer relationships.

## Data Availability

The raw data supporting the conclusions of this article will be made available by the authors, without undue reservation.
